# A current review on animal models of anti-asthmatic drugs screening

**DOI:** 10.3389/fphar.2025.1508460

**Published:** 2025-01-28

**Authors:** Shivam Singh, Sunita Kularia, Shivakshi Shukla, Mithilesh Singh, Manish Kumar, Ashish Kumar Sharma

**Affiliations:** 1 Department of Pharmacology, Nims Institute of Pharmacy, Nims University Rajasthan, Jaipur, Rajasthan, India; 2 Department of Pharmaceutical Chemistry, Nims Institute of Pharmacy, Nims University Rajasthan, Jaipur, India

**Keywords:** leukotrienes, hyperresponsiveness, inflammation, anaphylactic, microshock

## Abstract

Asthma is a chronic inflammatory respiratory condition characterised by airway constriction, smooth muscle spasm, and severe morbidity. It affects around 300 million people globally, with children being especially vulnerable. Despite its worldwide effect, the invention of innovative asthma medicines has been slow over the last 5 decades, leaving significant unmet requirements in asthma care. Although intriguing medicines have demonstrated efficacy in animal models, many fail to fulfil safety and effectiveness requirements in human trials, highlighting the critical need for more predictive models that better transfer to human results. This comprehensive review investigates the mechanisms and efficacy of anti-asthmatic drugs using both genetic and conventional animal models. Both genetic and traditional models of anti-asthmatic agents, their characteristics, and their significance are summarized as: *In-vitro* Models: Histamine receptor assay, Cell Culture Method, WST Assay, Spasmolytic Activity of the Lungs of Guinea Pigs, Airway and Vascular Responses to an Isolated Lung, The Isolated Perfused Guinea Pig Trachea’s Reactivity. *In-Vivo* Models: *In vivo* small animal models, Broncho Spasmolytic Activity in anaesthetized Guinea Pigs, Guinea Pigs Respiratory and Vascular Dysfunction Caused by Arachidonic Acid or platelet-activated factor (PAF), Guinea Pig Asphyxia Induced by Serotonin Aerosol and Anaphylactic Microshock, Guinea Pigs Under Anaesthesia: Histamine-Induced Bronchoconstriction, Microshock in Rabbits and Pneumotachography in Guinea Pigs, Guinea Pig Bronchial Hyperactivity, Guinea Pig Airway Microvascular Leakage, Mice With Inflammatory Airways. Conclusion: This review focusses on the benefits and limitations of current animal models in asthma research, emphasising the need for more sophisticated, predictive models to decrease translational failures. By critically evaluating these models, the review emphasises their importance in directing anti-asthmatic drug development and highlights the urgent need for innovation to bridge the gap between preclinical success and clinical efficacy.

## 1 Introduction

Obstructive airway disorders, such as asthma and chronic obstructive pulmonary disease (COPD), both are severe threats to global health. Asthma is the most frequent chronic condition. According to World Health Organization by 2030, COPD is predicted to surpass asthma as the third most common cause of death globally in children (Ribeiro-Silva et al., 2018; Mannino and Buist, 2007; Miles et al., 2012). Asthma and COPD share pathophysiological elements of airway inflammation, airway obstruction, and airway hyperresponsiveness (AHR). The main differences between the two conditions lie in the cellular and molecular characteristics of inflammation and the reversibility of airflow limitation. In allergic asthma, the airways are frequently infiltrated by eosinophils; in contrast, the primary cause of airway inflammation in COPD is neutrophils. However, in other non-allergic endotypes of asthma, such as exercise-induced, aspirin-sensitive, and infection-induced asthma, eosinophilic inflammation may be negligible or non-existent. Severe steroid-resistant and obesity-related asthma endotypes are often associated with neutrophilic inflammation (Lötvall et al., 2011). *Ex vivo* and *in vitro* models have been developed by scientists because the fundamental cause of asthma has historically been associated with a malfunction in the smooth muscle of the airways. These models allow them to investigate the factors that mediate changes in the function of the smooth muscle of the airways and how to treat them. The majority of characteristics of airway smooth muscle are presumably explained by the smooth muscle’s heightened sensitivity, force production, or shortened velocity in response to contractile agonists (Pyrgos et al., 2011). While investigations using the method of forced oscillation demonstrate that asthmatic RRS fluctuate over time, these changes could be the result of fleeting smooth muscle contractions in the airways (Lall et al., 2007). Because of this, the majority of patients responded well to inhaled beta-agonists, which relax airway smooth muscle, as a suggested treatment for acute symptoms. Nonetheless, concerns have been raised about the direct significance of airway smooth muscle contraction in light of the growing theory that asthma is a disease of remodelling of the airway wall. Various physical and biological changes, such as thickening of the airway wall, (Pare et al., 2007), enlargement of the group of smooth muscles surrounding the airways, (James et al., 2012), change in the composition of the extracellular matrix (ECM), (Araujo et al., 2008), infiltration mediated by myofibroblasts (Dolhnikoff et al., 2009) inflammatory cell infiltration, (Laurent et al., 2008)^,^ (Barnes, 2008) and epithelial dysfunction are examples of remodeling that can lead to airway hyperresponsiveness (Holgate, 2007). Importantly, some features of airway wall remodeling may precede clinical symptoms, thus contributing to the reduction of airway-lung interdependence and the loss of bronchodilation induced by deep inspiration (Baldwin and Roche, 2002).

## 2 Anti-Asthmatic drug screening models

### 2.1 *In vitro* models

#### 2.1.1 Histamine receptor assay

This technique assesses a test compounds affinity for the histamine H^1^ receptor by assessing their inhibitory actions on the binding of 3H pyrilamine (an H1 antagonist) to a preparation of the brain plasma membrane from guinea pigs. A 300–600 g male guinea pig is sacrificed through CO_2_ necrosis. After homogenisation of the brain in an ice-cold Tris solution (pH 7.5, 1 g in 30 mL of buffer), the homogenate is centrifuged at 50,000 g for 10 min at 4°C. After removing the supernatant, the pellet remains suspended in the buffer and centrifuged again. Following centrifugation, the pellet is reconstituted in 1 mL aliquots and stored at −70°C in Tris buffer (1g/5 mL). A shaking bath at a constant temperature of 25°C is used to incubate 50 µL of pyrilamine 3H (2 × 10^−9^ M), 50 µL of test compound (10^−5^–10^−10^ M), and 100 µL of membrane suspension from brain (10 mg/mL) of guinea pigs in each sample for 30 min. Tris HCl solution (50 mM, pH 7.5) is used as the incubation buffer. Saturation examinations has been performed using 11 conc. of 3H Pyrilamine (0.1–50 × 10^−9^ M). In the presence of incubation buffer, total binding is measured; non-specific binding occurs in the presence of mepyramine (10^−5^ M). The glass fibre filter stops the reaction with a quick vacuum filtration. Following that, the radioactivity is isolated from the membrane-bound material. After adding 3 mL of cocktail or scintillation sample to a liquid scintillation counter, the radioactivity retained on the filter due to the binding of the membrane is measured (Hill et al., 1978). Numbers such as percentage inhibition of pyrilamine 3H binding (defined as 100% of the control value) and total pyrilamine 3H binding, nonspecific binding, and special binding (total ligament-non-specific ligament) are determined. The IC_50_ value is the dissociation constant (K_i_) of the test substance obtained using the computer-assisted analysis of binding data from the experiment that evaluated 3H-pyrilamine in an unorganised drug (Hill et al., 1978). Limitations: Although the histamine H1 receptor assay is still a useful method for studying receptor-ligand interaction, its drawbacks emphasise the importance of cautious experimental planning and result interpretation. Complementary strategies, such as functional assays, *in silico* modelling, and human cell-based systems, must be incorporated into the process in order to overcome these difficulties.

#### 2.1.2 Cell culture method

An innovative experimental system called CULTEX technology is designed to cultivate and expose cells to ultrafine particles, gases, or fixed-flow combinations of both on an intermittent basis at the air/liquid interface. Because it is challenging to expose cultured cells directly to air pollutants such as gaseous or particulate chemicals and complicated mixes. Studies on the cytotoxicity of these substances have usually relied on animal trials. The efficiency of *in vitro* research has risen mainly to this method, which also permits direct exposure of the bronchial epithelial cells. The CULTEX technique exposes complex mixes directly at liquid/air interface, such as sidestream cigarette smoke, using a transwell membrane technology. The bronchial epithelial cells are moved from the companion plate to the cell exposure unit and then cleaned with PBS before being exposed. Human bronchial epithelial cells are susceptible to several parameters, such as gas flow rate and duration of exposure. After studying these factors, the cells are exposed to either clean air or varied amounts of side stream smoke for an hour. An exposure top that has been particularly made and fitted to the CULTEX machine can enable direct exposure, resulting in a uniform aerosol distribution above the cell cultures Cells exposed to the test environment and the air/liquid controls undergo identical incubation times and preparation for the analysis. The bronchial epithelial cells in the test group are exposed to varying quantities of side stream smoke for an hour after having been injected with the test medication for 24 h (Knebel et al., 2002). The number of cells, metabolic activity, and glutathione concentration are the biological characteristics that can be assessed for the treatment and control groups Additionally, the WST assay and electronic cell counting can be used to determine the cell viability in test and control groups (Piperi et al., 2003). Limitations: For researching how air pollutants affect bronchial epithelial cells at the air/liquid interface, CULTEX technology offers a potent platform. However, in order to fill in the gaps in reality and adaptability, the limits mentioned above highlight the necessity of meticulous experimental design, protocol standardisation, and complementing strategies. A more thorough grasp of the toxicological effects of air pollutants can be attained by researchers by integrating the CULTEX approach with sophisticated multicellular systems, *in vivo* models, and computer simulations.

#### 2.1.3 WST assay

In the WST assay, cells are transferred from exposure vessels to companion plates containing 2 mL of fresh RPMI medium per well. Then, 500 µL of medium with 100 µL of WST-1 dye is added to the attached cells, and after 1 h of incubation, it is removed. Next, 100 µL aliquots are transferred to a 96-well microplate for absorbance measurement at 450 nm/630 nm using a microplate reader. Additional cells from the same membrane are treated with 500 µL of trypsin/EDTA to detach them from the monolayer. After 4 min of incubation at 37°C, the enzymatic activity is stopped by adding 25 µL of trypsin inhibitor. The cells are gently suspended and diluted in CASYton, and 100 µL aliquots are analysed using an electronic cell counter. The CULTEX system allows bronchial epithelial cells to be exposed to airborne substances, providing a platform for *in vitro* toxicological evaluations of inhaled compounds. This method introduces new strategies for testing the toxicity of various air pollutants. Limitations: The CULTEX system, in conjunction with the WST assay, provides important information about the cytotoxic effects of airborne pollutants. However, because of its limitations, a thorough and accurate toxicological assessment requires careful experimental design, strict controls, and supplementary assays. This method’s limitations can be addressed, and its usefulness in inhalation toxicity research increased by combining it with more sophisticated approaches like co-culture systems or omics-based analysis.

#### 2.1.4 Spasmolytic activity in Guinea pig lungs

Several autacoids, such as histamine and leukotrienes, cause bronchoconstriction. Histamine induces bronchoconstriction by activating H1 receptors and is a key mediator in allergic and inflammatory responses. Calcium ionophores trigger leukotriene release through the 5-lipoxygenase pathway, leading to strong bronchoconstriction. This method tests drugs for their ability to inhibit bronchospasm caused by histamine or calcium ionophore. Albino guinea pigs, weighing between 300 and 450 g, are euthanized using an ether overdose. Their lungs are removed, cut into 5 cm strips, and placed in a physiological saline solution. The lung strips are mounted in an organ bath filled with a nutritive solution, which contains NaCl, NaHCO_3_, KCl, CaCl_2_, MgCl_2_, NaH_2_PO_4_, Na_2_HPO_4_, glucose (5%), and has a pH of 8. The bath is bubbled with carbogen and kept at 37°C. The tissue is preloaded with 0.5 g–3 g and allowed to equilibrate for 30–60 min. Before testing, carbachol is added to confirm the lung strip’s contractility. Two baseline values are recorded after administering the spasmogen (such as histamine dihydrochloride, calcium ionophore, or leukotrienes (C4 and D4) and measuring the maximal contractile response. After a 20 min equilibration, the spasmogen is administered again, followed by the test compound 5 min later. The contractile response is measured isometrically, and the percentage inhibition of spasmogen-induced contraction by the test drug is calculated. Limitations: When researching bronchospasm and assessing spasmolytic medications, the guinea pig lung spasmolytic activity assay is still a useful method. In order to overcome its drawbacks, its limits emphasise the necessity of cautious experimental design, ethical considerations, and supplementary techniques. Humanised systems, sophisticated *in vitro* models, or computer simulations could improve the assay’s results’ dependability and applicability.

#### 2.1.5 Vascular and airway responses to the isolated lung

This model allows for the simultaneous measurement of pulmonary vascular and airway responses to drugs. Sprague Dawley rats, weighing 300–350 g are anesthetized with an intraperitoneal injection of pentobarbitone sodium (50 mg/kg). After cannulating the trachea, the animal is placed on artificial respiration. The rat is given 1,000 units of heparin, and blood is rapidly drawn from the carotid artery to induce exsanguination. Following a median sternotomy, the lung is exposed, and a ligature is tied around the aorta to prevent systemic blood loss. The lung is then removed and suspended in a water-jacketed chamber, kept at 39°C and 100% humidity. The pulmonary artery is catheterized, and a heat exchanger regulates the temperature of the Krebs-Henseleit perfusion solution, which is continuously stirred in a reservoir. Using a peristaltic roller pump, the lungs are perfused at a flow rate of 8–14 mL/min to maintain a pulmonary arterial pressure of 15 mmHg. Pulmonary arterial pressure, airway pressure, and reservoir blood levels are continuously monitored, averaged electronically, and recorded using a polygraph. The effects of test compounds on pulmonary arterial and airway pressures are measured in mmHg and compared to baseline levels (Allen et al., 1993). Limitations: A useful tool for researching pulmonary vascular and airway responses in a controlled environment is the isolated lung model. Nevertheless, its drawbacks highlight how crucial additional methods-like sophisticated *in vitro* systems, *in vivo* models, and computer simulations are to improving the findings applicability and translatability. To optimise this model’s usefulness in pulmonary research, these limitations must be carefully taken into account throughout experimental design and interpretation.

#### 2.1.6 Reactivity of the isolated perfused Guinea pig trachea

This model is ideal for studying how the epithelium influences the reactivity of tracheal smooth muscle. It can be used to investigate the effects of substances such as histamine, calcium ionophores, bradykinin, leukotrienes, and potassium channel openers. Contractile agents can be applied to either the outer (serosal) or inner (mucosal) surface of the trachea. Albino guinea pigs of either sex, weighing between 300 and 550 g, are euthanized using CO_2_ narcosis. The entire trachea is excised, and individual rings are cut, with 12–15 rings tied together using silk thread. These rings are mounted in an organ bath containing Krebs-Henseleit buffer solution, which is pumped at a flow rate of 30 mL/min through the tracheal lumen. The tissue is kept at 37°C under a 0.5 g tension and is aerated with carbogen. Tracheal muscle responses are measured by monitoring the pressure difference between inlet and outlet catheters, which are connected to opposite sides of a differential transducer. Isometric contractions are recorded with a transducer linked to a polygraph. After a 45-min equilibration period, spasmogens are added to the organ bath. Commonly used spasmogens include carbachol (2 × 10⁻⁷ g/mL), histamine dihydrochloride (10⁻⁷ g/mL), calcium ionophore (10⁻⁶ g/mL), and leukotrienes C4 and D4 (10⁻⁸ g/mL). Once maximum contraction (initial spasm) is reached, a standard bronchodilator drug, such as isoprenaline (1 ng/mL) or aminophylline (10 ng/mL), is administered. The bronchial responses are recorded after reaching a stable plateau. The tissue is then washed thoroughly, and control contractions are induced again by adding a spasmogen. After reinducing the initial contraction, the test drug is added, and the contractile force is recorded at its peak. A 15 min washing interval is observed between experiments. Responses are quantified as changes in water pressure. EC50 values are calculated from dose-response curves using least square analysis and presented with 95% confidence intervals (Allen et al., 1993). The ED50 values can be determined, and the percent inhibition of spasmogen-induced contractions is also calculated (Baersch and Frölich, 1996). Limitations: Airway pharmacology and the effects of bronchodilators can still be studied using the isolated perfused guinea pig trachea model. Nonetheless, its limitations need for meticulous standardisation, experimental design, and result interpretation. Complementary methods, including sophisticated *in vitro* systems, computational models, and *in vivo* experiments, should be incorporated into research workflows in order to overcome these obstacles.

### 2.2 *In vivo* models

#### 2.2.1 *In vivo* small animal models

An animal model of asthma can only be considered legitimate and relevant if it can accurately replicate important aspects of the pathophysiology and symptoms of the human condition. Disease symptoms can be summarised in some ways, although every model is missing some components (Wanner, 1990; Krug and Rabe, 2008; Bates et al., 2009; Holmes et al., 2011). Rodents (Meurs et al., 2006; Lundblad et al., 2007; Kucharewicz et al., 2008)^,^ dogs (Stephens et al., 1986), lambs (Koumoundouros et al., 2006), pigs (Turner et al., 2002), horses (Herszberg et al., 2006) and non-human primates have all been employed as models to investigate ASM contraction or inflammatory responses (Table 1; Ayanoglu et al., 2011). Rodents are generally employed in pre-clinical and discovery research because they are inexpensive to maintain, can induce an “asthmatic” state through genetic manipulation or allergen challenge, and have short gestation periods. Despite this, their relevance for evaluating lung mechanics is questionable due to their size and high respiratory rate. Rats exhibit a number of characteristics of lung physiology and airway inflammation, such as early and late asthmatic reactions following an allergen exposure, that are comparable to human allergic asthma (Eidelman et al., 1988). Rats may be more useful for studying inflammation and immunology than for studying ASM contraction, though, since they have relatively modest bronchoconstrictor responses and their mast cells emit more 5-HT than histamine (Kucharewicz et al., 2008). In terms of the anatomy and pathophysiology of the airway and lungs, guinea pigs and humans are quite similar. This includes the release of histamine and leukotrienes from mast cells as well as the existence of both early and late asthmatic responses. The receptor pharmacology of guinea pig lungs mimics that of humans, potentially explaining the similarity of responses upon drug exposure (Ressmeyer et al., 2006). Although both humans and guinea pigs have axonal reflexes that enhance their exposure to allergen challenge, these reflexes are stronger in guinea pigs (Meurs et al., 2006; Canning and Chou, 2008) and a major disadvantage resides in a lack of separate natural and transgenic guinea pig strains. The most popular models of allergic asthma are those derived from mice, whose immune systems and genomes are highly characterised and thus allow for systematic manipulations of immune cell types through genetic engineering, pharmacological targeting, or adoptive transfer of particular immune cell subsets. Among rodent models, mice also have the shortest gestation period and the lowest cost. As a result, accurate assessment of illness endpoints and advanced lung function measuring methods are needed (Irvin and Bates, 2003; Corry and Irvin, 2006). AHR is frequently used as the primary metric in animal models; however, this may not always distinguish between contributing variables, such as EAR versus LAR, and characteristics of airway remodelling. Nevertheless, animal models are always being developed to provide more knowledge regarding disease pathophysiology. For example, the ability to distinguish between the separate impacts of airway inflammation and remodelling on lung function is being made possible by the development of chronic allergen exposure protocols that more successfully induce airway remodelling (Cates et al., 2004; Humbles et al., 2004; Johnson et al., 2004). Furthermore, there is an increasing understanding that, in comparison to more widely used ovalbumin models, utilising clinically relevant allergens, such as house dust mite, offers more tractable insight regarding human disease (Johnson et al., 2004; Lloyd, 2007). This has served as the foundation for study into the effects of combination treatments, exposure to various antigens, and even avoiding some allergens in order to replicate clinical settings (Fattouh et al., 2005; DiGiovanni et al., 2009). In asthmatic models, airway inflammation and hyperreactivity are generally associated with acute changes, whereas airway wall remodelling is linked to chronic changes. For instance, airway remodelling continues 9 weeks after the HDM challenge is stopped, whereas airway inflammation decreases but AHR only slightly decreases (Johnson et al., 2004). This implies that in the absence of inflammation, remodelling establishes structural characteristics that support hyperresponsiveness (Bates et al., 2009; Lloyd, 2007).

**TABLE 1:** An overview of popular models for ASM contraction research.

S.No.	Model	Merits	Demerits
1	*In vivo* small animal models	Evaluation of the combined function of distal organs Many techniques for creating hyper-responsive models	ASM contraction cannot be directly observed. Uncertain mechanical significance Airway wall remodelling might not be replicated
2	*Ex vivo* airway segments	Maintains the airway wall’s functioning mechanism. retains integrated multicellular functions One can quickly apply mechanical tension	ASM contraction cannot be directly observed limited to brief courses only
3	Thin cut lung slices	Maintaining parenchymal mechanics through direct visualisation of ASM contraction and signalling events maintains integrated multi-cell capacities	Breaking through diffusion barriers It is challenging to identify the mediators’ source and target
4	ASM strips/rings	ASM contractile function’s most direct measurement Simple mechanical strain application	Unable to examine integrated replies limited to studies with short to medium duration
5	*In vitro* cell culture	Extensive study is made possible by paying close attention to contractile regulatory pathways without relying on new tissue	Progressive phenotypic loss of the cell Absence of well-rounded replies mechanical environment that is not physiological
6	Engineered tissues	Rebuild tissues with individual cells Manage cell operations with distinct matrices	Models are largely unproven

The degree to which different cell types are integrated and the intricacy of the mechanical environment dictate the specific benefits and drawbacks of each mode.

#### 2.2.2 Broncho spasmolytic activity in anesthetized Guinea pigs

Using this technique, alterations to a living animal air volume inside a closed system made up of the trachea, bronchi, and respiratory pump are recorded. Additionally, a reservoir which allows the measurement of the pressure or volume of extra air might be used. Bronchoconstriction causes an increase in the volume of surplus air and a decrease in the volume of inspired air. The administration of spasmogen is the outcome of bronchial smooth muscle contraction. By measuring the amount of air that the lung does not take in following a bronchospasm, the approach allows for the evaluation of the broncho spasmolytic action. Guinea pigs weighing between 250 and 500 g is anaesthetised intraperitoneally with 1.25 g/kg urethane to prevent spontaneous respiration. The trachea is cannulated; a Statham P23 Db transducer is attached to one arm, and a breathing pump is connected to the other. A frequency of 60 strokes per minute has been employed to artificially ventilate the animal. A polygraph measures and records excess air that is not inhaled by the lungs. To give test drugs and assess blood pressure, cannulations are inserted in the jugular vein and carotid artery. A 15-L plastic container is used for each animal. Spraying is done with an aerosol of 0.25% histamine solution at 180 mmHg pressure. Five minutes is the exposure period. One hour prior to the exposure, the test medication is taken orally. The spasmogen test is given once more. Animals with no protection suffocate on their sides. The resulting percent reduction of induced bronchospasm over the control agonistic responses is reported as ED50 (Miura et al., 1994). Limitations: The model of broncho spasmolytic activity in anaesthetised guinea pigs is still a useful method for researching acute bronchodilation and constriction. However, because of its limits, supplementary methodologies, ethical approval, and careful consideration of experimental design are required. Other models, such as sophisticated *in vitro* systems or computer simulations, ought to be investigated in order to overcome these drawbacks and improve the findings’ applicability and universality.

#### 2.2.3 Arachidonic acid or PAF-induced respiratory and vascular dysfunction in Guinea pigs

Prostacyclin and thromboxane are byproducts of the metabolism of arachidonic acid. Propranolol induces thrombocytopenia and bronchoconstriction, while prostacyclin lowers diastolic and systolic blood pressure. Intraperitoneally, male guinea pigs weighing 300–600 g are sedated with 60 mg/kg pentobarbitone sodium. A cannulated jugular vein is used to administer the spasmogen/test compound. Both of the carotid arteries have cannulations; one is used to withdraw blood and the other is attached to a pressure transducer to monitor blood pressure. There is a respirator attached to the trachea 70 to 75 strokes/min. Not absorbed by the lungs, excess air is carried to a transducer equipped with a broncho timer, which converts variations in airflow into an electrical signal. Airflow variations and arterial blood pressure variations are continuously monitored. Animals are given numerous intravenous injections of the same arachidonic acid dose 60 μg/kg until they experience two equally intense bronchospasms. Both the spasmogen and the test chemicals are injected intravenously. Calculations are made between the pre-drug treatment contra values and the percent inhibition or rise of thrombocytopenia, haematocrit, blood pressure decrease, and bronchospasm following test drug administration. The duration and the amount of blood pressure lowering are established (Lefort and Vargaftig, 1978). Limitations: The guinea pig model of arachidonic acid or PAF-induced respiratory and vascular dysfunction is a helpful method for researching vascular reactions and acute bronchospasm. But because of its drawbacks, careful experimental planning, moral defence, and the employment of complementing alternative models are required. By combining this model with human-based methods, computer simulations, or *in vitro* research, the results can become more pertinent and useful.

#### 2.2.4 Anaphylactic microshock in Guinea pigs

Several locations release histamine during the anaphylactic reaction. Antihistamines are useful in treating a variety of anaphylactic symptoms, including serum sickness and bronchial asthma. Microshock may take place when an alien protein enters the body. It appears that a microshock is a repetitive shock that is interrupted prior to death. Guinea pigs weighing 200–300 g is become more sensitive by injecting egg albumin subcutaneously. The animals are exposed to a 5% albumin aerosol in an exposure chamber following a 3-week period. The moment they start to have dyspnoea, they are taken out of the chamber. They will perish if removed at that very moment. As a gauge of the shock’s intensity, the preconvulsion time and interval between the start of exposure to acute dyspnea is recorded. The animal is considered protected and the pre-convulsion period presumed to be endless when, within 6 min, no evidence of shock is observed. The degree of protection (p) is calculated from the formula:

$$p=\left[1-\left(C/T\right)\right]\times 100$$

C and T are pre-convulsion time of the control and treated animals respectively. A medication will be given to the guinea pig following a control exposure. It undergoes another 4–7 days of exposure to a control (Chand et al., 1994; Vitkun et al., 1990). Excluded animals are with C less than 40 s and greater than 165 s. Limitations: The anaphylactic microshock model in guinea pigs offers important information about the effectiveness of antihistamines and the immediate consequences of anaphylaxis. Nevertheless, its drawbacks underscore the necessity of meticulous experimental planning, moral deliberation, and the supplementary use of substitute models. This model can improve the translational value of research findings when combined with *in vitro* systems, computer simulations, or human-relevant methodologies.

#### 2.2.5 Serotonin aerosol-induced asphyxia in Guinea pig

When guinea pigs are exposed to a serotonin-containing aerosol, their bronchi contract, which may result in suffocation if the constriction is sufficiently severe. 200–300 g guinea pigs are put in an anaesthetic box, and a compressor is employed to inject 2% serotonin aerosol. Animals exposed to the aerosol exhibit distinctive behaviour and gradually exhibit respiratory difficulties, convulsions, and even death. Through observation, one can build experience in accurately judging the pre-convulsion time, which remains rather constant if the guinea pigs are not used frequently. The animals are brought outside as soon as the proconvulsive breathing starts. Within a minute, an infusion pump injects the test medication. The percent protection afforded by the drug is calculated from the formula (1-T_1_/T_2_) ×100 where T_1_, is the mean of the control preconvulsion time 2 days before, and 2 days after the administration of the drug and T_2_, is the preconvulsion time determined with the administration of the drug (De Bie et al., 1998; Konno et al., 1993). Limitations: In order to assess medications that target bronchoconstriction, especially those intended to relieve acute respiratory distress, the serotonin aerosol-induced asphyxia in guinea pig’s paradigm is helpful. The model’s acute nature, lack of systemic data, species-specific responses, ethical problems, reproducibility issues, and emphasis on serotonin as a single mediator rather than the more intricate, multiple causes of human respiratory disorders are some of its drawbacks. The results should be evaluated with caution, as with any animal model, and supported by more studies, such as clinical trials, to ensure that they are applicable to human illnesses.

#### 2.2.6 Histamine-induced bronchoconstriction in anesthetized Guinea pigs

A plethysmograph can be used to measure respiratory parameters in guinea pigs, such as respiratory frequency and respiratory amplitude. Following inhalation of histamine, bronchodilatory medications reduce the reflectory increase in respiratory frequency and the fall in respiratory amplitude. A catheter placed into the pleural cavity and a Fleisch tube can be used to record additional breathing data. Moreover, the bronchodilator effects of potassium channel openers or antagonism against bradykinin-induced bronchoconstriction can be assessed using this approach. Both sexes of 400–600 g Guinea pigs are anaesthetised intraperitoneally with 70 mg/kg pentobarbital, and their jugular vein, carotid artery, and trachea are cannulated. The animal is kept alive with artificial breathing (60 breaths per min). With tracheal, venous, and arterial catheters attached to onset ports in the plethysmograph box wall, the guinea pigs are housed inside a whole-body plethysmograph box. Connected to the respirator is the tracheal port. A differential pressure transducer is utilised to measure the airflow rate into and out of the plethysmograph. Measured quantities include tidal volume and transpulmonary pressure. To calculate pulmonary resistance (PR) and dynamic lung compliance (LC), signals from the airflow, tidal volume, and transpulmonary pressure are sent into an online computer system. Using a Statham pressure transducer, systemic arterial pressure is determined. Pain pulses are used to calculate heart rate. When 0.5–2.0 μg/kg of histamine is injected intravenously, baseline values of LC and PR are increased by 200%. Challenges are repeated after a 5-min interval, producing the same rise in pulmonary resistance over the course of the experiment. The test chemical is injected intravenously 1 min prior to the administration of histamine following three consistent responses. The test compounds inhibition of histamine-induced bronchoconstriction is noted, and the ED_50_ is computed. In addition, the duration of histamine antagonistic action is assessed (Vitkun et al., 1990).

#### 2.2.7 Pneumotachography in Guinea pigs

**FIGURE 1:**
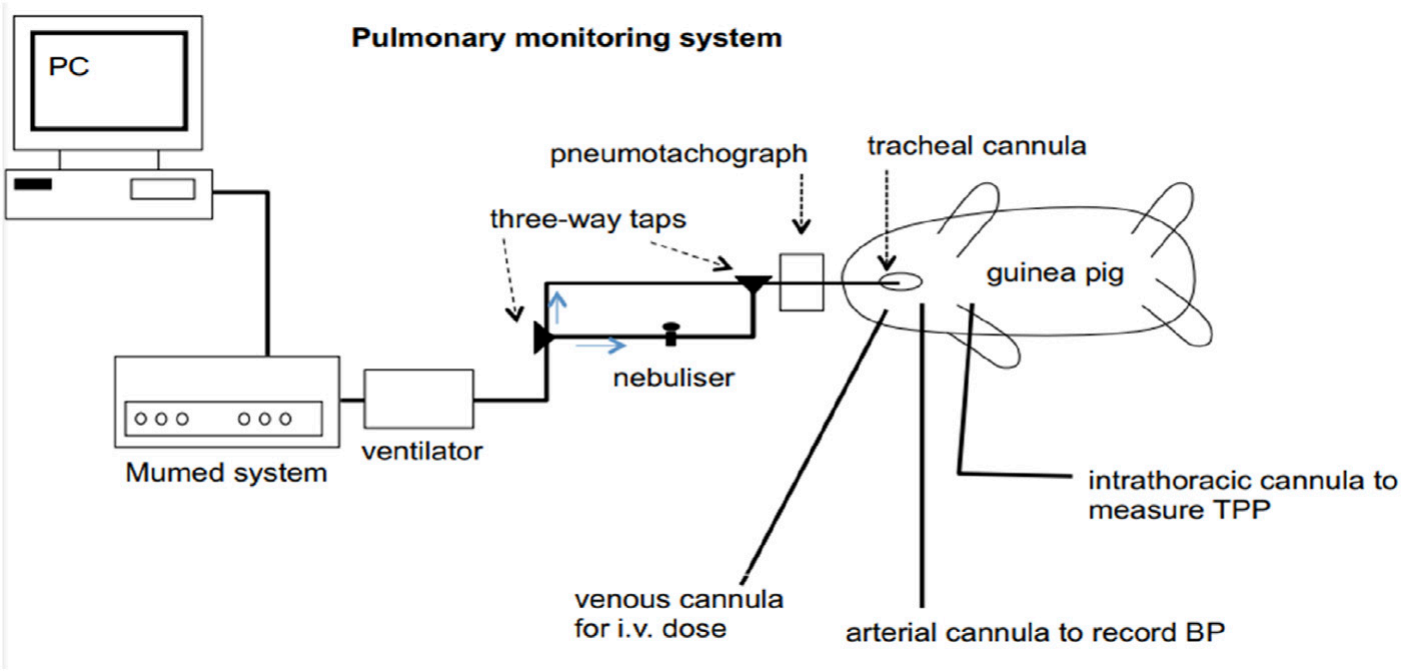
Diagrammatic representation of the combined experimental system for guinea pig pulmonary function testing, nebulised aerosol administration, and breathing. TPP (transpulmonary pressure) and BP (blood pressure).

In guinea pigs under anaesthesia, pneumotachography allows the simultaneous evaluation of several parameters of respiration and blood circulation. The Fleisch tube serves as the basis for its application. 1.5 g/kg urethane is administered intraperitoneally to anaesthetise 300–400 g guinea pigs. On a warm surgical table, the upper limbs of the animal are fixed. The cannula is inserted into the trachea. To measure intrathoracic pressure, a thin plastic catheter is placed in the oesophagus with the point in the chest. Also, the cannula is inserted into the carotid artery on one side and the cephalic vein on the other. The pneumotachograph is attached to the tracheal cannula. The differential pressure transducer is attached to the pneumotachograph. An additional differential pressure sensor is isolated from ambient air and connected to the oesophageal catheter. An instrument called a Gould pressure sensor is used to record blood pressure. An oscilloscope is used to monitor oesophageal pressure and airflow signals. Many respiratory and circulatory parameters are measured using an analogue computer. It is possible to measure the mechanics of the lung, such as the final work of breathing, the dynamic compliance, and the resistance of the airways. Each animal acts as its master (Figure 1). The results from the 5 min before the initial administration of the substance are evaluated and used as controls for each individual process. After the administration of the chemical, the response values are presented as a percentage of the control (Kleinstiver and Eyre, 1979). Limitations: A few drawbacks of the pneumotachography model in guinea pigs are the artificial settings (such as mechanical ventilation) in which the animal is kept, the invasive nature of the operation, and the use of anaesthesia, which inhibits normal respiratory function and reflexes. Additionally, the model mainly assesses mechanical elements of lung function, which might not accurately reflect the intricate, multifaceted nature of infections involving the immune system.

#### 2.2.8 Microshock in rabbits

Due to its sensitivity to histamine, the guinea pig has been the preferred model for screening antihistaminic drugs; however, when a second species is sought, the rabbit may be an appropriate substitute. A glass tank of 61 × 30.5 × 30.5 cm, with its open side down and a hole linked to a nebuliser, is filled with 200–300 g of rabbits. Every rabbit is housed in a different chamber and exposed to a 0.2% histamine aerosol. When given in modest doses, rabbits gradually show signs of shock. The drawing in of the abdomen walls to facilitate breathing is the initial symptom. The respiration either increases faster and shallower or slower and deeper, and the movements get stronger. In the subsequent scenario, the animal’s head swiftly oscillates “to and fro.” It is necessary to remove the animal from the chamber at this point. In the event that this is not the case, the symptoms appear quickly afterward and may include breathing difficulties, seizures, cyanosis, urine, and even death. There must be at least 8 days gap between testing to avoid desensitisation. Half an hour before the experiment, the test medication is injected intraperitoneally. A time stamp is used to document when the animal must be taken out of the chamber (Ali et al., 1994; Ali et al., 1992; Harris et al., 1976). The percent protection offered by the drug is calculated using the formula

$$\left(1-T_{1}/T_{2}\right)\;x\;100$$

Where, T_1_ = control preconvulsion time.

T_2_ = preconvulsion time after administration of the drug.

Limitations: There are a number of drawbacks to using the rabbit microshock model to test antihistaminic medications, such as species-specific physiological variations, possible histamine-responsive variability, and the acute character of the reaction. The model ignores the intricate, multi-mediator mechanisms underlying human allergic reactions and shock states, instead concentrating only on histamine-induced shock. The therapeutic usage of these medications in humans, especially for chronic illnesses, may not be fully reflected by the short-term evaluation of drug efficacy and the use of intraperitoneal drug delivery. The interpretation of results is further complicated by the possibility of desensitisation with repeated testing, animal heterogeneity, and confounding effects brought on by stress.

#### 2.2.9 Bronchial hyperactivity in Guinea pigs

Histamine or other spasmogen inhalation can cause guinea pigs to have symptoms similar to bronchial asthma, such as asphyctic convulsions. Using an ultrasonic nebuliser, the challenging substances are applied as aerosols. Anaphylactic convulsions are the last sign, followed by increased breathing frequency and forceful inspiration. The onset of these symptoms may be postponed by antagonistic medications. The preconvulsion time is quantifiable. Albino guinea pigs weighing 300–400 g has been used in this activity Three boxes, each with a 1.5 L/min airflow, make up the inhalation cages. The animal is put into box A, where the standard or test medication is administered using an ultrasonic nebuliser. An aerosol of the test drug solution 0.2 mL is given via an infusion pump in less than a min. The test medication or the standard is administered subcutaneously or orally to the animal as an alternative. The animal is transferred into Box C via Box B, which acts as a sluice. There, an ultrasonic nebuliser is used to expose the guinea pig to an aerosol of a 0.1% solution of histamine hydrochloride. Amount of time is measured till asphyctic convulsions appear. The animal is then quickly taken out of the inhalation box. Calculations are made to determine the percentage increase in preconvulsion time compared to controls and the ED_50_, or 50% increase in pre-convulsive time (Rogers et al., 1989). Limitations: This guinea pig model of bronchial hyperactivity has a number of drawbacks. The concentration on acute reactions leaves out chronic inflammatory elements of the disease, and species-specific characteristics restrict its relevance to human asthma. Stress from handling and moving animals between cages can have an impact on outcomes, and causing asphyctic convulsions presents serious moral questions. We ignore other important asthma-related metrics, such as inflammation and airway remodelling, in favour of measuring preconvulsion time. Consistency is further complicated by differences in drug administration methods and difficulties standardising aerosol delivery. Additionally, possible variability resulting from sex or weight differences is overlooked when only male guinea pigs within a particular weight range are used. Desensitisation may result from repeated histamine exposure, and the model’s applicability for larger asthma research is limited by the lack of thorough outcome indicators such as cytokine levels. These elements draw attention to issues with reproducibility, dependability, and translational significance.

#### 2.2.10 Airway microvascular leakage in Guinea pigs

Evans *in vivo* research on plasma exudation in guinea pig airways can be conducted using blue dye. This approach can be used to investigate the antagonistic effects of bradykinin and platelet-activating factor (PAP) on microvascular leakage and vagal stimulation-induced airway responses. Urethane (1.5 g/kg, intraperitoneally) is used to sedate guinea pigs weighing between 380 and 600 g. The carotid arteries, jugular vein, and trachea are cannulated. Blood pressure is measured via carotid artery catheterisation and test substance delivery via jugular vein cannulation. The animal is kept on artificial respiration at a rate of 60 breaths per min. Throughout the experiment, lung resistance is measured as a gauge of airway function and observed. A pressure transducer, with one end connected to the catheter placed into the right pleural cavity and the other to the intratracheal cannula, is used to measure transpulmonary pressure. A pneumotachograph is used to measure airflow. The injectable test substance is administered. Evans Blue dye is administered intravenously for 1 min after 10 min. Bradykinin, platelet-activated factor (PAF), or vagal stimulation injection causes bronchoconstriction and microvascular leakage after 1 min. The thoracic cavity is opened for 6 min after the induction of leakage, and a ventriculostomy is used to implant a cannula into the aorta. To remove the intravascular dye from the systemic circulation, perfusion is carried out using 100 mL (0.9%) saline at a pressure of 100–120 mmHg. After opening the ventricle, 30 mL of saline are perfused inside. Moreover, the lungs are removed. The tissues are weighed after being blotted dry. Evans Blue dye is extracted in 2 mL of formamide, and its wavelength is determined at 620 nm using a spectrophotometer. Lung resistance and Evans Blue dye concentration has been measured as ng/mg tissue, and are compared statistically between treated groups and controls that merely received the challenge (Aoki et al., 1988; Chand et al., 1992; Perretti and Manzini, 1993).

#### 2.2.11 Airway inflammation in mice

**FIGURE 2:**
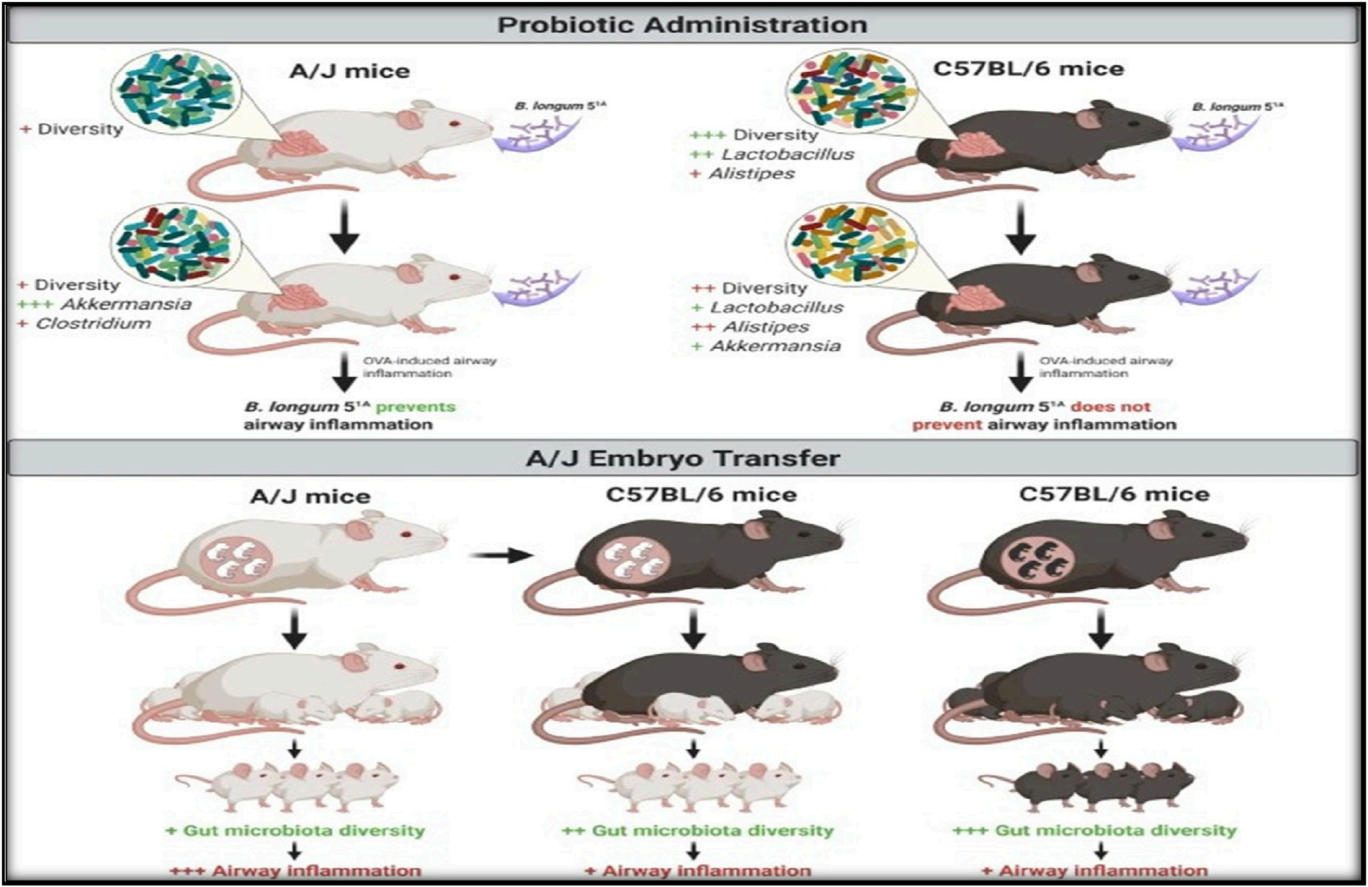
Diagram illustrating the impact of embryo transfer and Bifidobacterium longum 51A treatment on experimental allergic airway illness in A/J and C57BL/6.

In bronchoalveolar lavage (BAL) fluid, ovalbumin-sensitised Balb/c mice exhibit significant eosinophilia when exposed to ovalbumin repeatedly. Eosinophil inflow has also been observed to differ significantly amongst mice stained differently. A low-responder or non-responder strain would be 129/SV or CBA. Eosinophil counts in the BAL and lung tissue significantly rise in response to antigen challenge in strains like SWR, FVB, and C57BL/6. For this investigation, heat-coagulated egg white is subcutaneously implanted into Balb/c mice (Mitchell et al., 1999; Waserman et al., 1995). After 14 days, mice get an intratracheal challenge with heat-aggregated ovalbumin. Oral, intraperitoneal, or subcutaneous delivery of the test drug is feasible. Animals from both groups had their bronchoalveolar lavage fluid collected 48 h after the antigen challenge, and the total number of eosinophils, neutrophils, and eosinophil peroxidase activity were measured. After the animals are killed, a histological analysis is done (Figure 2). The protection provided by the test drug is assessed in light of the findings of the histopathological analysis and the BAL fluid evaluation (Hessel et al., 1995; Temann et al., 1998).

## 3 Preclinical models of asthma

Numerous alterations observed in people can be successfully modelled in small animals by inducing them. Although certain animals are more likely than others to experience allergic reactions in the wild, laboratory animals cannot naturally develop allergic asthma; instead, the response needs to be purposefully generated (Slauson and Hahn, 1980; Kurucz and Szelenyi, 2006). Allergen-induced asthma-like changes in the classical model, animals are first made more sensitive to a particular antigen, ovalbumin, and then they are given an adjuvant usually Al(OH)_3_, though some have also used ricin and *Bordetella* pertussis) to help specifically prime the Th2 immune response. Generally, the antigen/adjuvant are given to the animal suspension once or twice to sensitise them systemically (by subcutaneous or intraperitoneal administration). Two weeks later, they are exposed to allergens (challenge) in the airways (generally by aerosol delivery). To elicit the desired reaction, different species, strains, and research organisations may use different concentrations, durations, and frequencies of sensitisations and challenges. For instance, compared to the protocols designed for guinea pigs, the problems in rat models typically involve larger allergen concentrations and longer durations (Kips et al., 1992; Misawa and Chiba, 1993; Renzi et al., 1996; Schneider et al., 1997). All of the primary symptoms of allergic asthmatic response, including IgE production, acute bronchospasm, oedema, mucus formation, Th2 inflammation, eosinophilia, neutrophilia, a LAR (bronchoconstriction), and nonspecific AHR, are successfully induced by the ovalbumin model. These reactions, however, differ throughout species, strains, techniques, and labs. In this model, for instance, the allergic response in rats is dependent on IgE, (Nonaka et al., 2000), but in guinea pigs, it is dependent on IgG1 (Nonaka et al., 2000; Riedel et al., 1996). Depending on the procedure followed, it may rely on IgE or IgG1 or be somewhat independent of allergen-specific immunoglobulin in mice (Hamelmann et al., 1999; Crosby et al., 2002; Korsgren et al., 1997; Hamelmann et al., 1997). There are various benefits to employing guinea pigs, despite the fact that there is a notable distinction between the responses in people and guinea pigs, where the response is IgE-driven. Both, in humans and guinea pigs, histamine and leukotrienes play a major role in controlling acute bronchospasm; in mice, however, serotonin and LTD4 are involved (Canning, 2003; Szelenyi, 2000; Hele et al., 2001). In actuality, compared to rats or Guinea pigs, the existence of a real EAR in mice is less certain. Second, despite the fact that guinea pigs also have sympathetic innervation, which is absent from human airways, both humans and guinea pigs have cholinergic parasympathetic and noncholinergic relaxing innervation of the airway smooth muscle, and they respond to bronchoconstrictors in a similar manner. However, in mice and rats, there is no noncholinergic (i.e., relaxing) innervation of the smooth muscle (Szelenyi, 2000; Hele et al., 2001; Zosky et al., 2006; Canning and Fischer, 2001; Skloot et al., 1995). Furthermore, it has been proposed that large dosages of these medications are required to induce a physiological shift in airway function because the smooth muscle of rats and mice is frequently less susceptible to bronchoconstrictors than that of guinea pigs or humans (Martin et al., 1988; Laberge et al., 1995; Haczku et al., 1996). Third, guinea pigs are more anatomically related to humans than the majority of other tiny animal species. They feature identical lobular divisions of the lung, a dichotomous airway-branching pattern, and well-developed bronchial smooth muscle. Mice and rats have different lung lobular divisions on the left and right, and their airways are asymmetrical (Patra, 1986). Another way that animal allergy reactions differ from one another is through modelling the LAR. Given that it has been accurately measured by several groups for modelling the late-phase, the rat is likely the best animal (Renzi et al., 1993; Rabb et al., 1994). The LAR has been measured in guinea pigs by various research groups, however the results show significant variation. It appears that whether a LAR is seen depends on the techniques employed for sensitising and challenging the animals (Selig et al., 2006). In contrast, most researchers struggle to induce a LAR in mice, although a few have successfully done so. Despite these differences, a common finding across studies is that allergens trigger a strong inflammatory response in guinea pigs, mice, and rats, similar to what occurs in human asthma. However, the severity of the response varies across different strains of rats and mice used in these studies (Zosky et al., 2006; De Bie et al., 2000). This strain-specific variation provides a unique chance to investigate potential pathways underlying sensitivity or resistance to allergic responses. Because there are fewer strains available for guinea pigs than for mice and rats, the choices they have are actually limited. Since they have been shown to react to allergens more strongly than other strains, the Brown Norway rat and BALB/c mouse are the most widely used strains (Hylkema et al., 2002; Trifilieff et al., 2000). Other frequently utilised mouse strains are A/J mice, which are well-known for having innate airway hyperresponsiveness (AHR), and C57BL/6 mice, which are employed since a lot of genetically modified (GM) mice were produced from this strain (Levitt and Mitzner, 1989). These models show inflammatory responses that are similar to those found in human asthma, such as an increase in neutrophils, eosinophils, and CD4^+^ Th2 cells, as well as higher lavage fluid levels of the cytokines IL-4, IL-5, and IL-13. Furthermore, these models respond to standard asthma treatments, such as β-agonists and glucocorticoids (Selig et al., 2006). While these models share many similarities with human asthma responses, certain limitations must be noted. The fact that chemokine and cytokine activity can vary between species is a major problem. For example, IL-13 can cause human B cells to change their IgE class but not mouse B cells (Lai and Mosmann, 1999). Species-specific variations in chemokine receptors can result in variable compound efficiency, which presents another difficulty when evaluating chemokine antagonists intended for the human receptor in mice. Before exploring chemokine antagonists *in vivo*, these concerns should be addressed using *in vitro* models that are specific to each species (Owen, 2001; Proudfoot, 2002). Concerns with eosinophils in these models also exist. For instance, naive Brown Norway rats are vulnerable to spontaneous eosinophilic granulomatous pneumonia, while naive Guinea pigs may display airway eosinophilia (Rothenberg et al., 1995; Noritake et al., 2007). Furthermore, whether mouse eosinophils can degranulate *in vivo* exactly the same manner as human eosinophils are a topic of continuous discussion. These species-specific variations imply that, in contrast to humans, other processes may be underlying the AHR linked to the allergic reaction in mice (Hessel et al., 1995; Lefort et al., 1996; Persson et al., 1997; Lacy et al., 2001). Data from a human trial, however, cast doubt on the eosinophil’s function in the allergic/asthmatic response, as they showed that monoclonal antibodies directed against IL-5 were efficient at lowering eosinophils in lavage fluid but had no effect on other asthma-related symptoms, such as AHR. Airway eosinophil levels and AHR were also found to be independent in studies using antiIL-5 antibodies in mouse and guinea pig allergen models (Corry et al., 1996; Mauser et al., 1993).However, divergent results from research employing two genetically modified, ∆dbl GATA mice and eosinophil-deficient mouse strains-PHIL have left the role of the eosinophil in the allergic response up for question. In contrast with the human anti-IL-5 study, allergen exposure shielded PHIL animals of allergen-induced AHR (Lee et al., 2004), but it enhanced AHR in ∆dbl GATA mice to levels comparable to wild-type controls (Humbles et al., 2004), corroborating results from the human trial. Given that ∆dbl GATA mice were bred onto a BALB/C background and the PHIL mice were bred onto a B6 background strain differences could account for the disparities observed in these two investigations. The concept that strain variations may account for these inconsistent results is supported by studies utilising mice defective for IL-5 bred onto either a BALB/C or B6 (Foster et al., 1996; Hogan et al., 1998) and showing differences in AHR following exposure to allergen (B6 protected, while BALB/C remaining hyperresponsive). It had been proposed that this issue could be tackled by crossing these lines onto the opposite background strain (Wills-Karp and Karp, 2004). Genetic background can impact the efficacy of any treatment, which can have implications for the successful development of any medication in a polygenic disorder such as asthma. These statistics do, however, underline this point. It has been demonstrated that AHR occurs in all three species following antigen sensitisation and challenge. Once more, there can be differences in species, strains, methods, and labs when it comes to the existence and intensity of AHR. For example, the guinea pig lung, exhibits seasonal fluctuation and is highly susceptible to nonallergic airway hyper-reactivity, which may obscure findings (Ressmeyer et al., 2006; Underwood et al., 2002). This may imply that, even with the same strain of rats, the animals employed by the various labs differ in ways that are intrinsic or environmental and may have an impact on the reactions results. The main physiological alteration that these models measure in mice is AHR A/J mice (Cates et al., 2004) and high IgE-producing mice (Eum et al., 1995) exhibit the most prominent AHR, which can differ depending on the sensitization/challenge regimen (Zosky et al., 2004).

## 4 New allergen models

In an attempt to mimic clinical situations more accurately, new disease-relevant antigens have been utilised recently, including ragweed, cockroaches, *Ascaris suum*, Aspergillus fumigatus, and Dermatophagoides farinae (house dust mite). Furthermore, as individuals may get sensitised by repeated intranasal sensitisations rather than systemic ones, techniques utilising this approach have been created (Temelkovski et al., 1998; Johnson et al., 2004). Sensitisation and prolonged intranasal administration of low-dose house dust mite extracts, for instance, have been shown to cause eosinophilia, long-term airway remodelling, and acute heart failure (AHR) (up to 9 weeks). Another technique developed to lessen the use of adjuvant is the adoptive transfer of dendritic cells and T cells into the severe combined immunodeficiency (SCID) mouse using human peripheral blood mononuclear cells (hPBMCs) from house dust mite-sensitive patients (pulsed and primed *in vitro*). This can further remove the model from the clinical situation (Duez et al., 2000). However, these models have additional limitations that are outside the purview of this review. Using chronic allergen exposure methods to construct models is another trend.

These models have two specific goals:

- to simulate the long-term alterations in airway remodelling that asthmatics experience; and
- to develop models that more closely resemble real-world clinical situations by allowing drugs to be tested under therapeutic dosage regimens i.e., in which inflammation exists prior to the compound’s administration).

Different labs have had varying degrees of success in replicating these modifications, but unlike asthma, the remodelling generated in many of these models is more likely to be the product of fibrosis than smooth muscle thickening (Zosky and Sly, 2007; Selig et al., 2006). Much of the problem is assumed to stem from mice, rats, and guinea pigs developing immune resistance to increasing allergen assaults, which prevents them from reproducing these long-term changes. To circumvent these problems, many organisations have started sensitising younger animals, whose immune systems are still maturing (Fulkerson et al., 2005; Ostroukhova et al., 2004; Melkild et al., 2002). Once more, this is an attempt to replicate the clinical setting, since it is believed that modifications to the immune system during early life (and development) affect an individual’s propensity to acquire asthma. It will be crucial for developing these unique chronic models that are clinically relevant in order to discover new asthma treatments. It will also be necessary to develop a steroid-resistant model of allergen-induced inflammation. These models, which are similar to those with COPD, are required to represent the disease pathways linked to a subgroup of asthmatics who are not sensitive to steroid treatment (You et al., 2006).

## 5 Innovation in model development

The lack of translation from preclinical to clinical research of novel asthma chemicals and biologics is especially concerning for individuals with severe and/or therapy-resistant asthma, for whom new and effective medications are necessary for enhanced asthma management. More accurate representations of the asthmatic airway are required for safety and effectiveness testing, target validation, and mechanistic study. An innovative, cooperative, and multidisciplinary approach will be required to address this.

### 5.1 Biomimetic models

The possibilities provided by tissue engineering, along with improvements in bioreactor and scaffold design, have led to the availability of tissue-engineered human airway counterparts in a variety of forms. While these have yielded valuable insights into the pathophysiology of the disease, they remain too primitive to faithfully mimic important *in vivo* features, such as a functional immune or circulatory system (Nichols and Cortiella, 2008).

### 5.2 Microfluidics

Tissue engineering is an area that is rapidly expanding, and its practitioners have responded to the challenge of modelling complexity. While complete immune or circulatory system simulation *in vitro* may still require some work, progress is being made. This is where tissue engineering comes into its own, together with advancements in the quickly expanding field of microfluidic lab-on-a-chip technology. The liver, kidney, brain, muscles, and blood vessels have all been successfully microfabricated via microfluidics to aid in basic research and medication development. In comparison to current macroscopic methods, it also has a number of benefits (Song et al., 2009).

### 5.3 *In silico* modelling

Methods for mathematical and *in silico* modeling may also shed light on the fundamental causes of asthma. By incorporating data from genomic, proteomic, physiological, environmental, and behavioural methods into mathematical models that depict the mechanistic foundations of disease pathophysiology, researchers can investigate the connection between cellular and whole-organ responses in a different way. The biomedical research community has been comparatively sluggish to adopt *in silico* modelling compared to other industries like the automotive or aerospace sectors. Recent reports from the FDA and IMI acknowledge the potential of *in silico* technology to alleviate bottlenecks in the drug discovery process and lower the total cost of drug research, so this could change (Anon, 2006). Every step of the drug development and discovery process can benefit from the use of *in silico* modelling. Several companies are changing the way they create medications and speeding up the drug discovery process by investing heavily *in silico* modelling (Van De Waterbeemd and Gifford, 2003). The current generation of modelling tools for asthma research and medication development is being formed by these techniques. Several models have been created to investigate fundamental phenomena, including whole-organ functions (such bronchoconstriction and aerosolised drug deposition in the lung), molecular interactions (ligand–receptor), and even virtual patients. Organisations are working with academic and industrial life scientists to create virtual patients for a variety of illnesses, such as respiratory, metabolic, and cardiovascular conditions (Epstein, 2004; Huang et al., 2009).

In the context of a disease or therapeutic area, technological tools can replicate the complexity of human biology, with an emphasis on understanding the biological mechanisms, pathways, and feedback loops that underlie illness progression as well as assessing the clinical response to possible treatments (Musante et al., 2002). According to reports, a respiratory module can recapitulate the biology of asthma and can replicate the disease in various patient types (e.g., adults with established chronic disease; patients who respond to treatment and those who do not; mild, moderate, or severe asthmatics) in order to understand patient heterogeneity, something that is not achievable with current *in vivo* approaches. To expedite their asthma medication discovery procedures, a number of pharmaceutical companies, including Pfizer, Merck, Sanofi Aventis, and Bayer, are already utilising this technology platform. This strategy is thought to have saved Aventis (now Sanofi Aventis) millions of dollars by identifying interleukin (IL)-5 as an unfeasible target for human asthma, despite encouraging preclinical data in animals. As a result, Aventis ceased creating treatments that targeted this cytokine (Lewis et al., 2001). Other companies that are currently conducting clinical trials on asthmatic patients have verified this outcome (Flood-Page et al., 2007; Kips et al., 2003; Leckie et al., 2000). Mepolizumab, a blocking monoclonal antibody that targets IL-5, may be helpful in a very small percentage of people with asthma (<0.5%), according to current research. A medication called mepolizumab has shown great effectiveness in treating some hypereosinophilic disorders (Schwartz et al., 2010; Kim et al., 2010). And in exacerbations of asthma in patients with severe illness who had chronic eosinophilia in their sputum while taking large doses of oral and inhaled corticosteroids. (Gleich, 2009; Pavord et al., 2010) Efficacy against this asthma outcome measure was not expected, which is not surprising given that exacerbations have not been modelled in in silico models. It is probable that these *in silico* models will be employed earlier in the drug development process to weed out inappropriate substances prior to preclinical investigations as trust in the technology increases with regular use. Throughout the pharmaceutical industry, these systems biology techniques are being used extensively as supplements to conventional preclinical *in vitro* and *in vivo* models. At this point, it is unlikely that they will take the place of animal testing, but with more efficient early screening techniques, it is possible that animal testing for candidate molecules that will be discontinued later in development will be decreased, as is the case with IL-5. According to the IMI, the creation of *in silico* techniques is one of two “Individual Research Projects of Priority” that must be addressed right away by a future European Centre of Drug Safety Research. This underscores the technology’s potential significance in facilitating the quicker discovery and development of better medications with less animal testing. Although the usage of *in silico* modelling is growing, its acceptance is being slowed by a number of problems. These include a shortage of scientists with the specialised knowledge and abilities needed to develop these models (mathematicians, engineers, and informaticians), as well as the requirement for computers and software with the capacity to handle the growing amount of data needed. Adopting these strategies is further complicated by the fact that *in silico* procedures depend on the precision and scope of the current body of knowledge to be filled in. Although the models will reflect any gaps in our comprehension of the disease’s causes, experts have suggested that the technology would never fully replace conventional approaches to drug discovery and development (Bel et al., 2011).

### 5.4 Precision cut lung slices

*Ex vivo* human lung precision-cut lung slices (PCLS) provide exciting opportunities for our further understanding of the genesis and course of asthma and act as a bridge between animal experimentation and the culture of cells and isolated tissue preparations. Nevertheless, despite this, PCLS approaches are not frequently employed. The study of airway disease has greatly benefitted from PCLS, which was initially developed for toxicological research and has several advantages over current *in vivo* techniques. Their main use has been to assess the responses of the smooth muscle of the airways to different stimuli, including drugs, allergens, and infections; these responses might include hyperresponsiveness, remodelling, and bronchoconstriction (Ressmeyer et al., 2006).

### 5.5 Transgenic approaches

Transgenic approaches: In fundamental research, genetically engineered animals—particularly mice—are regarded as crucial models for identifying certain pathways for drug development, mechanistic studies, target identification, and/or validation. Many “off-the-shelf” transgenic mice and species-specific probes and reagents are commercially available and have been widely employed in asthma research. These tools allow researchers to selectively inhibit, turn off, or upregulate a single molecular signalling pathway. The value of these models for researching a disease linked to many molecular and cellular pathways that either work in concert or independently of one another is debatable, though. Transgenic mice have demonstrated the significance of several cytokines, such as eotaxin, IL-4, IL-5, IL-9, IL-13, and regulated upon activation, normal T cell expressed and secreted (RANTES), in Th2-driven inflammatory responses in pulmonary inflammation (Kaiko and Foster, 2011). Although these studies have contributed to the analysis of the pathophysiology of allergic asthma, they have not yet demonstrated their use in comprehending asthma brought on by environmental and lifestyle variables. Suppressing or upregulating a single molecular route is unlikely to affect asthma because it is caused by a multitude of variables, as demonstrated for IL-4, IL5, and IL-13 (Leckie et al., 2000; Borish et al., 2001; Corren et al., 2010; Oh et al., 2010). However, there is debate on whether this is because of inadequate evidence for the significance of these cytokines in human asthma (as opposed to animal models of allergic-type inflammation) or poor clinical trial study design (i.e., participants not reflecting asthma heterogeneity) (Trusheim et al., 2007; Corren et al., 2010; Borish, 2010). It is evident that results from any of these genetically altered models must be interpreted in light of the complexity of human asthma rather than being regarded in a vacuum. In an attempt to simulate this complexity, transgenic mice with double and triple genes are being created.

By selectively turning gene expression on or off at any time, this gives researchers models for identifying various signals and components that comprise particular pathways, enabling the definition of the reversibility of phenotypic responses (Elias et al., 2003; Lee et al., 2006; Ochkur et al., 2007). However, when creating transgenic models, the same limitations that apply to traditional animal models (such as strain and species variations and sensitisation techniques) also exist, making it even more crucial that animal research is reported truthfully so that the results can be understood correctly. Although preliminary findings indicate that the data should be interpreted cautiously, it is still too early to determine if transgenic models are helpful predictors of efficacy in humans, even though there is little doubt that they will provide insight into the pathobiology of disease (Kilkenny et al., 2010).

## 6 Conclusion

Animal models are critical for advancing asthma research, yet each model presents specific advantages and limitations. Given the multifactorial nature of asthma, no single animal model can fully replicate the complexities of human asthma. A variety of species, including dogs, sheep, pigs, ferrets, hamsters, rabbits, mice, rats, guinea pigs, horses, and nonhuman primates, have been utilized, each contributing valuable insights but also carrying the risk of incomplete or misleading extrapolation to human outcomes. It is essential to recognize that animal models are proxies, not perfect representations of human biology. Therefore, findings from these models should always be interpreted in conjunction with human data to ensure greater relevance and accuracy.

The key challenge lies in the fact that no one model can definitively determine the therapeutic potential of novel compounds. However, when used judiciously, well-designed animal models can provide critical information about drug effects and mechanisms. To address current limitations, the integration of emerging technologies such as tissue engineering, imaging, and *in silico* modeling holds great promise. These advancements can help create more predictive models that minimize the use of animals while improving the reliability of results.

Incorporating these innovative tools into preclinical testing will be essential to refining current research methods, reducing animal use, and ultimately expediting the development of effective asthma therapies. Such a shift will not only improve model accuracy but also help bridge the gap between preclinical research and clinical outcomes, facilitating the development of more effective and targeted treatments for asthma.
