# Correction: A current review on animal models of anti-asthmatic drugs screening

**DOI:** 10.3389/fphar.2025.1675265

**Published:** 2025-11-13

**Authors:** Shivam Singh, Sunita Kularia, Shivakshi Shukla, Mithilesh Singh, Manish Kumar, Ashish Kumar Sharma

**Affiliations:** 1 Department of Pharmacology, Nims Institute of Pharmacy, Nims University Rajasthan, Jaipur, Rajasthan, India; 2 Department of Pharmaceutical Chemistry, Nims Institute of Pharmacy, Nims University Rajasthan, Jaipur, India

**Keywords:** leukotrienes, hyperresponsiveness, inflammation, anaphylactic, microshock

In the **Abstract**, the phrase “*In-vitro* Animal Models” was erroneously used in place of “*In-vitro* Models”.

A correction has been made to the **Abstract**:

“Both genetic and traditional models of anti-asthmatic agents, their characteristics, and their significance are summarized as: *In-vitro* Models: Histamine receptor assay, Cell Culture Method, WST Assay, Spasmolytic Activity of the Lungs of Guinea Pigs, Airway and Vascular Responses to an Isolated Lung, The Isolated Perfused Guinea Pig Trachea’s Reactivity.”

In the **Introduction**, *subheading 2.1* was incorrectly called “*In vitro* animal models” in place of “*In vitro* models.”

*Subheading 2.1* has now been corrected as “2.1 *In vitro* models” in section **Introduction**, 2 Anti‐Asthmatic drug screening models, 2.1 *In vitro* models.

The original article has been updated.

